# Inverse correlation between serum interleukin-6 and iron levels among Japanese adults: a cross-sectional study

**DOI:** 10.1186/2052-1839-14-6

**Published:** 2014-02-28

**Authors:** Hiroko Nakagawa, Takashi Tamura, Yoko Mitsuda, Yasuyuki Goto, Yoshikazu Kamiya, Takaaki Kondo, Kenji Wakai, Nobuyuki Hamajima

**Affiliations:** 1 Department of Preventive Medicine, Nagoya University Graduate School of Medicine, 65 Tsurumai-cho, Showa-ku, Nagoya 466-8550, Japan; 2 Department of Hematology and Oncology, National Hospital Organization, Higashi Nagoya National Hospital, Nagoya, Japan; 3 Department of Pathophysiological Laboratory Sciences, Nagoya University Graduate School of Medicine, Nagoya, Japan; 4 Department of Healthcare Administration, Nagoya University Graduate School of Medicine, Nagoya, Japan

**Keywords:** Serum interleukin-6, Serum iron, Inflammation, *Helicobacter pylori*

## Abstract

**Background:**

Interleukin-6 (IL-6) is a multifunctional cytokine that is produced by many different cell types, and plays an important role in the regulation of inflammation, immune responses, the acute-phase response, and hematopoiesis. Previous laboratory and clinical studies have shown that IL-6 causes a significant decrease in serum iron levels. Therefore, we conducted an epidemiological study to examine the association between serum IL-6 and iron levels.

**Methods:**

In total, 280 Japanese individuals aged 20–78 years were enrolled when they visited a clinic located in an urban area for *Helicobacter pylori (H. pylori)* infection tests and subsequent eradication; 65.3% were infected with *H. pylori*. Subjects with gastric cancer, idiopathic thrombocytopenia, or IL-6 > 10 pg/mL were excluded from the study. Serum iron and IL-6 levels were measured using the 2-nitroso-5-(N-propyl-3-sulfopropylamino) phenol method and chemiluminescence enzyme immunoassay, respectively.

**Results:**

Geometric mean iron and IL-6 levels were 111.5 μg/dL and 1.77 pg/mL, respectively, for men, and 89.4 μg/dL and 1.55 pg/mL, respectively, for women. The logarithm of serum iron levels was negatively correlated with the logarithm of IL-6 levels in men (r = −0.19, p = 0.047), but not in women (r = −0.035, p = 0.65). Regression analysis, adjusted for sex, age, and *H. pylori* infection status, showed that the logarithm of serum iron levels was significantly associated with a decreased logarithm of IL-6 levels (β = −0.053, p = 0.041). The odds ratio for low serum iron levels adjusted for sex, age, and *H. pylori* infection status was 7.88 (95% CI 1.29–48.06) in those with an IL-6 level > 4 pg/mL.

**Conclusion:**

Lower serum iron levels are significantly associated with higher serum IL-6 levels among Japanese adults.

## Background

Interleukin-6 (IL-6) is a multifunctional cytokine that is produced by many different cell types, including monocytes, lymphocytes, fibroblasts, endothelial cells, keratinocytes, mesangial cells, and endometrial cells [1,2]. IL-6 plays an important role in the regulation of inflammation, immune responses, the acute-phase response, and hematopoiesis [3]. IL-6 exerts its effects at the systemic and local tissue level, and across a wide range of cell types [4,5].

Hepcidin, a liver-produced peptide hormone, was first reported in 2000 [6-9]. Hepcidin is the main regulator of body iron homeostasis and acts as an antimicrobial peptide by limiting iron availability [10]. Hepcidin synthesis is regulated by body iron status and is induced by IL-6 during inflammation [11,12]. IL-6 is required to induce hepcidin, and the IL-6-hepcidin axis is responsible for hypoferremia caused by inflammation [11]. Some laboratory studies have shown a relationship between IL-6, hepcidin, and serum iron levels [11-13], and this relationship was subsequently shown in clinical studies [14-19]. However, most of these previous studies have been conducted in specific settings with particular patients or groups. Few epidemiological studies have reported any association between serum IL-6 and iron levels. Therefore, this cross-sectional study aimed to examine the association between IL-6 and serum iron levels among Japanese adults.

## Methods

### Subjects

The subjects were Japanese adults who visited an urban clinic in Nagoya, Japan, between December 2005 and October 2010 to be tested for *Helicobacter pylori (H. pylori)* infection and, if positive, to receive treatment. They were apparently healthy individuals who were concerned about possible *H. pylori* infection. During this period, we enrolled 280 individuals at the clinic (105 men and 175 women) aged 20–78 years. Subjects with gastric cancer or idiopathic thrombocytopenia were excluded, as were those with IL-6 > 10 pg/mL (n = 4). All subjects gave written informed consent to take part in this study. The study was approved by the Ethics Committee of Nagoya University School of Medicine (approval number 155).

### Clinical tests

All participants provided blood samples in the morning. No fasting was required. Serum iron levels and total iron binding capacity (TIBC) were measured using the 2-nitroso-5-(N-propyl-3-sulfopropylamino) phenol method, and IL-6 levels were determined by chemiluminescence enzyme immunoassay. The coefficient of variability of serum iron assay was 1.57%. Transferrin saturation (TSAT) (%) was calculated as serum iron ÷ TIBC × 100. The reference range for serum iron was 54–200 μg/dL in men and 48–154 μg/dl in women, and that for serum IL-6 was less than 4 pg/mL for both sexes. We determined the cutoff level for high serum IL-6 levels among apparently healthy Japanese people (50 men and 49 women), corresponding to the mean + 1.96 × SD. Low levels of serum iron were defined as <54 μg/dL in men and <48 μg/dL in women. We also determined the cutoff level for low serum iron among apparently healthy Japanese people (52 men and 36 women) corresponding to the mean – 1.96 × SD, for both sexes. The reference range for serum TIBC was 253–364 μg/dL in men and 246–410 μg/dl in women. *H. pylori* infection was determined using the ^13^C-urea breath test; patients with a value ≥ 2.5% were considered to be infected.

### Statistical analysis

The log_10_-transformed values of serum iron and IL-6 showed a near-normal distribution, and therefore, they were used in the analysis. Age was classified into 10-year strata. The strength of the associations between the logarithm of serum iron levels, the logarithms of serum IL-6 levels, serum TIBC levels, and TSAT were examined using the Pearson correlation coefficient. Multivariate regression analysis was performed to assess the effect of log_10_ of IL-6 on log_10_ of serum iron, TIBC, and TSAT, with adjustment for sex and age (continuous variables). In addition, odds ratios (ORs) and 95% confidence intervals (CIs) for low serum iron were adjusted for sex, age (continuous variables), and *H. pylori* infection status. A two-sided p value of less than 0.05 was considered statistically significant. All statistical analyses were performed using Stata version 11.1 (STATA Corporation, College Station, TX, USA).

## Results

Table 1 shows subject characteristics according to sex. The subject’s mean (±SD) age was 53.1 ± 12.8 years (53.6 ± 13.8 years for men and 52.8 ± 12.2 years for women) and most were aged between 50–69 years for both sexes. Geometric mean serum iron levels were 111.5 μg/dL for men and 89.4 μg/dL for women. Two (1.9%) men and 13 (7.4%) women had low iron levels. Geometric mean IL-6 levels were 1.77 pg/mL for men, 1.55 pg/mL for women, and 1.63 pg/mL overall. Six (5.7%) men and six (3.4%) women were above the reference range of IL-6 (≥4 pg/mL). Those with an IL-6 level < 3.0 pg/mL accounted for 87.6% of men and 92.0% of women. Of the 268 subjects who underwent the ^13^C-urea breath test, 175 (65.3%) were infected with *H. pylori* based on the test results. The positive rate for *H. pylori* was 59.8% in men and 68.7% in women.

**Table 1:** Subjects’ characteristics and distribution of serum iron and interleukin-6 levels

**Characteristics**	**Males**		**Females**		**Total**	
	**n**	**%**	**n**	**%**	**n**	**%**
Age (years)						
20–29	5	4.8	6	3.4	11	3.9
30–39	15	14.3	25	14.3	40	14.3
40–49	17	16.2	31	17.7	48	17.1
50–59	25	23.8	50	28.6	75	26.8
60–69	37	35.2	56	32.0	93	33.2
70–79	6	5.7	7	4.0	13	4.7
Serum iron (μg/dL)						
<50	2	1.9	15	8.6	17	6.1
50–100	24	22.9	84	48.0	108	38.6
101–150	63	60.0	65	37.2	128	45.7
151–200	15	14.3	9	5.1	24	8.6
>200	1	0.95	2	1.1	3	1.1
Serum interleukin-6 (pg/mL)						
<1.0	14	13.3	25	14.3	39	13.9
1.0–1.9	49	46.7	99	56.6	148	52.9
2.0–2.9	29	27.6	37	21.1	66	23.6
3.0–3.9	7	6.7	8	4.6	15	5.4
>4.0	6	5.7	6	3.4	12	4.3
*Helicobacter pylori* infection						
Infected	61	58.1	114	65.1	175	62.5
Uninfected	41	39.0	52	29.7	93	33.2
Missing	3	2.9	9	5.2	12	4.3
Total	105	100	175	100	280	100

**Figure 1:**
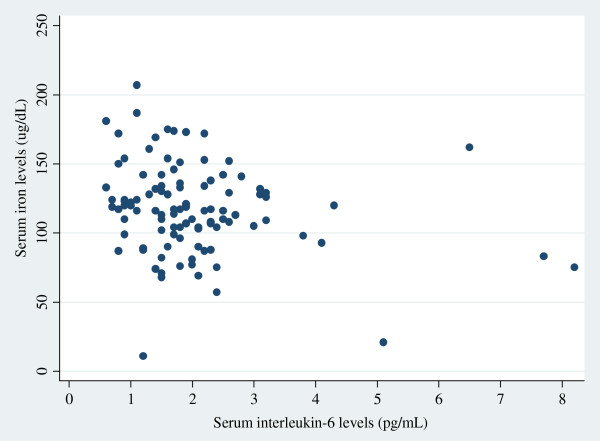
Correlation between serum interleukin-6 and iron levels among men (r = −0.19, p = 0.047, n = 105).

**Figure 2:**
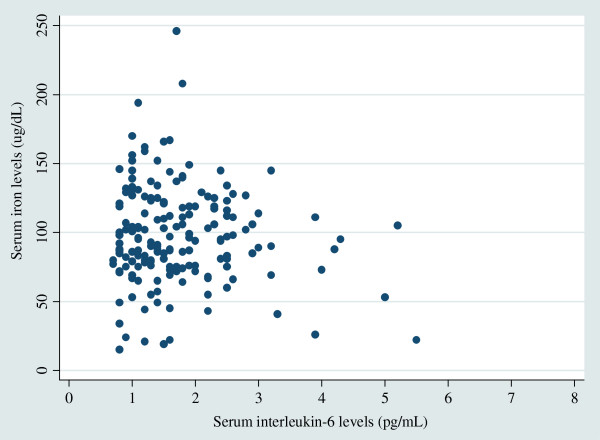
Correlation between serum interleukin-6 and iron levels among women (r = −0.035, p = 0.65, n = 175).

Figure 1 shows a scatter plot of serum IL-6 and iron levels for men, and Figure 2 shows a similar scatter plot for women. The logarithm of serum iron levels was significantly negatively correlated with the logarithm of IL-6 levels in men (r = −0.19, p = 0.047), but not in women (r = −0.035, p = 0.65), or in either sex (r = −0.054, p = 0.37). However, in multiple linear regression analysis on log_10_ serum iron adjusted for sex and age, lower log_10_ serum iron levels were significantly associated with higher log_10_ serum IL-6 levels in all subjects (β = −0.050, p = 0.040). However, this did not reach statistical significance in each sex; β = −0.059 in men (p = 0.073) and β = −0.048 in women (p = 0.17). Even when stratified by *H. pylori* infection status, the direction of association was not altered. Regression analysis, adjusted for sex, age, and *H. pylori* infection status, also showed that the logarithm of serum iron levels was inversely associated with the logarithm of IL-6 levels (β = −0.053, p = 0.041). The OR for low serum iron levels, adjusted for sex, age and *H. pylori* infection status, was 7.88 (95% CI 1.29–48.06) in those with an IL-6 level > 4 pg/mL (versus ≤ 4 pg/mL). In subjects with an IL-6 level ≥ 3 pg/mL (versus < 3 pg/mL), the OR for low serum iron levels, adjusted for sex, age and *H. pylori* infection status, was 8.21 (95% CI 2.0–34.3).

Mean serum TIBC levels were 305.7 μg/dL for men (range, 224–431 μg/dL) and 329.9 μg/dL for women (range, 209–501 μg/dL). A low TIBC level was found in 8.6% (n = 9) of men and in 1.14% (n = 2) of women. A high TIBC level was found in 12.4% (n = 14) of men and in 8% (n = 15) of women. Serum TIBC levels were not correlated with the logarithm of IL-6 levels overall (r = −0.106, p = 0.16). In multiple linear regression analysis adjusted for sex and age, serum TIBC levels were not significantly associated with log_10_ serum IL-6 levels in all subjects (β = 3.53, p = 0.58). Mean TSAT (%) was 39.4 for men (range, 3.0–71.1) and 30.5 for women (range, 3.7–77.6). TSAT (%) was not correlated with the logarithm of IL-6 levels overall (r = −0.036, p = 0.55). However, regression analysis adjusted for sex and age showed that a lower TSAT (%) was significantly associated with a higher logarithm of serum IL-6 levels in all subjects (β = −3.31, p = 0.040) and in men (β = −5.45, p = 0.028), but not in women (β = −1.93, p = 0.36).

## Discussion

In this cross-sectional study, lower serum iron levels were significantly associated with higher serum IL-6 levels among Japanese adults. To the best of our knowledge, this is the first epidemiological report to reveal an association between serum iron levels and serum IL-6 levels, even in an apparently healthy population.

Inflammation has a potent effect on iron homeostasis, by reducing intestinal iron absorption, and sequestering iron in macrophages, thereby decreasing serum iron levels [20]. There is now substantial evidence that these effects of inflammation are also mediated by hepcidin [20]. Hepcidin is a 25-amino acid peptide hormone secreted by hepatocytes that circulates in blood plasma and is excreted in the urine [7]. Hepcidin inhibits intestinal iron absorption, iron recycling in macrophages, and release of stored iron from hepatocytes, thereby decreasing body iron availability [10,19,21].

A relationship among IL-6, hepcidin, and iron has been shown in recent studies [11-14]. In human hepatocyte culture *in vitro*, hepcidin mRNA was dramatically induced by IL-6, but not by IL-1 or tumor necrosis factor-α [12]. A study conducted in human liver cell culture, mice, and human volunteers indicated that IL-6 alone can rapidly induce hepcidin synthesis and corresponding hypoferremia during inflammation [11]. Kemna et al. [14] determined temporal associations between IL-6, hepcidin, and serum iron levels in healthy individuals following injection of the inflammatory activator lipopolysaccharide. Lipopolysaccharide-induced elevation in circulating IL-6 levels was associated with increased urinary hepcidin levels and a subsequent significant decrease in serum iron levels [14]. In addition, there is evidence of IL-6-induced anemia [13]. Further evidence of this IL-6-induced anemia was provided by Nieken et al. [13], who examined oncology unit patients treated with recombinant IL-6 as an antitumor agent. IL-6 produced a rapid dilutional anemia in 3 days. At 4 weeks, serum iron levels decreased by 65% and mean cell volume slowly decreased, with a nadir at 6 weeks, which in conjunction with the hypoferremia, likely represents iron-restricted hematopoiesis. The anemia and hypoferremia were reversible after cessation of IL-6 [13]. In another study, metastatic renal cell carcinoma patients with IL-6 levels > 10 pg/mL had an increased risk of anemia (OR 3.86 p = 0.003) [22]. These findings show that IL-6 is an important cytokine that induces hypoferremia mediated by hepcidin. Our study showed that subjects with IL-6 levels > 4 pg/mL had an increased risk for low serum iron levels, even in a seemingly healthy population.

Some clinical studies have shown a positive correlation between hepcidin levels and serum IL-6 levels [15-18,22]. Among 60 chronic hemodialysis patients with and without chronic hepatitis C virus infection, serum prohepcidin levels were significantly correlated with serum IL-6 levels [15]. A positive correlation was found between serum hepcidin and IL-6 levels (r = 0.546, p = 0.023) in 17 patients with Crohn’s disease and anemia from this disease [22]. A randomized, double-blind crossover study among nine men showed a positive correlation between hepcidin and IL-6 immediately after prolonged exercise in the placebo arm of the trial [18]. Among 24 patients treated with pulmonary endarterectomy in deep hypothermic circulatory arrest, the maximum post-operative plasma levels of hepcidin were positively correlated with maximum IL-6 levels [17]. Furthermore, Kuo et al. [16] reported that serum hepcidin levels were negatively correlated with serum iron levels (r = −0.412, p = 0.002) among 86 patients with Kawasaki disease and 30 age-matched febrile controls. These findings led to the speculation that serum iron levels are negatively correlated with serum IL-6 levels. Although we could not measure blood hepcidin levels because of the limited samples and/or resources, our study supports this assumption that serum iron levels are negatively correlated with serum IL-6 levels. However, a few men in our study had high serum IL-6 levels, which might have led to an overestimation of the correlation. We found an inverse association between TSAT (%) and the logarithm of serum IL-6 levels in all subjects. However, this association varies between studies, and therefore, further investigations are required on this association. In 34 hemodialysis patients, serum IL-6 levels were not correlated with TSAT (r = −0.250, p = 0.154, n = 34) [23]. There was a marginally significant correlation between serum IL-6 levels and TSAT in 34 peritoneal dialysis patients (r = 0.067, p = 0.078) [24].

Recently, some reports showed an association between *H. pylori* infection and hepcidin levels [25-27]. Schwarz et al. reported [25] that gastric hepcidin expression was elevated during *H. pylori* infection, which normalized after successful treatment. However, serum hepcidin levels were not altered after infection or successful eradication [25]. Our result showing that *H. pylori* infection did not alter the association between serum iron and IL-6 levels is consistent with unaltered serum hepcidin levels of the above-mentioned study [25]. Our findings are also consistent with two previous studies that failed to find a relation between systemic hepcidin levels and *H. pylori* infection [26,27]. Therefore, we combined those subjects with *H. pylori* infection and those without infection in the analysis on the association between serum IL-6 and iron levels. In the same subjects as those in the current study, our previous study showed that serum ferritin levels were significantly lower in *H. pylori*-infected subjects than in uninfected subjects, but serum iron levels were unchanged [28]. Moreover, atrophic gastritis by *H. pylori* infection was associated with a decrease in ferritin levels, but not with serum iron levels. While ferritin levels represent the body’s iron store, iron levels remain stable because of homeostasis and most of the iron entering blood plasma comes from recycling. Therefore, we speculate that this decrease in serum ferritin levels is attributable to some extent by iron use by *H. pylori* or a reduction in intestinal iron absorption due to atrophic gastritis following infection. However, a decrease in serum iron levels has not been observed in our previous study.

A limitation of our study is that there was no information of dietary intake and of the actual time of bleeding from the subjects; both might have affected serum iron levels. Despite fluctuations in dietary iron intake and intermittent losses through bleeding, plasma iron levels in humans remain stable [29]. While most of the iron entering blood plasma comes from recycling, an appropriate amount of iron is absorbed from the diet to compensate for losses and to maintain nontoxic amounts in stores [29]. However, some studies have reported that diet has little effect on iron levels [30,31]. In addition, although hepcidin levels show diurnal variation, one study showed that the diurnal variation pattern was not affected by food intake [32]. Therefore, we believe that the lack of information of dietary intake did not considerably affect our findings of a correlation between serum iron and IL-6 levels. Because blood was drawn in the morning in all of the participants, the effect of diurnal variation in serum iron and hepcidin would be small. There are heritable differences in hepcidin expression that may determine phenotypic variation in iron metabolism between individuals. Therefore, consideration of phenotypic variation in iron metabolism in individuals is important. Another limitation of our study is that there were no data of phenotypic variation of hepcidin. Our study assessed the association between serum IL-6 levels and iron levels. Production of CRP is enhanced by IL-6 [33]. Assessment of other low grade inflammation markers such as high-sensitivity CRP, could be informative as an indication for the severity of infection, and this needs to be assessed in future studies.

## Conclusions

The present study shows that lower serum iron levels are significantly associated with higher serum IL-6 levels among Japanese adults. The OR for low iron levels is significantly increased in those with IL-6 levels > 4 pg/mL. Further studies are needed to determine the association between serum iron and IL-6 levels, taking into account serum hepcidin levels.

## Competing interests

The authors declare that they have no competing interests.

## Authors’ contributions

HN performed statistical analysis and drafted the manuscript. TT, YG, YK, and TK participated in recruiting subjects and collecting data. YM participated in collecting data and specimen management. KW helped to draft the manuscript. NH conceived the study, participated in recruiting subjects and collecting data, and helped to draft the manuscript. All authors read and approved the final manuscript.

## Pre-publication history

The pre-publication history for this paper can be accessed here:

http://www.biomedcentral.com/2052-1839/14/6/prepub
